# Molluscan Shell Matrix Characterization by Preparative SDS-PAGE

**DOI:** 10.1100/tsw.2003.30

**Published:** 2003-05-05

**Authors:** Frederic Marin

**Affiliations:** IsoTis, NV., ProfBronkhorstlaan, 10, Gebouw D, 3723 MB Bilthoven, The Netherlands

**Keywords:** biomineralization, molluscan shell, calcium carbonate, calcifying matrix, polydispersity, denaturing preparative SDS-PAGE, polyclonal antibodies, dot-blot, dialysis, yophilization

## Abstract

The glycoproteinaceous constituents of molluscan shell matrices usually resist chromatographical fractionation. We describe a protocol that overcomes this difficulty and permits collection of a large amount of shell proteins for further in vitro characterization. After dissolution of the mineral phase, the glycoproteins are fractionated “blind” on a preparative electrophoresis. They are subsequently detected with a polyclonal antibody raised against the whole matrix.

## INTRODUCTION

The secretion of molluscan shell is finely regulated by a complex array of glycoproteins, polysaccharides, and chitin, which self-assemble during the calcification process and stay entrapped within the shell[1]. These shell constituents exert a strict control on the nucleation and growth of calcium carbonate crystals. By terminating crystallization, they also determine the final shape of the crystals. Because of these multiple roles, they can be used in different ways, i.e., as constituents of biomimetic organomineral materials with superior mechanical properties[2,3] and as natural biodegradable antiscalant additives[4]. At last, they offer promising possibilities in tissue engineering and bone reconstruction[5,6].

However, due to their polydispersity, their polyanionic properties, and their glycosylation, these proteins usually resist classical chromatographical fractionation, stain very poorly on polyacrylamide gels, and absorb very little at 280 nm[7]. A consequence of these technical obstacles is that only few shell proteins are known at present[8], and their exact function during calcification remains elusive.

A way to improve the detection of shell matrix components consists of raising polyclonal antibodies against the nonfractionated matrix, and in using these antibodies on western-blots. Whatever the epitopes (proteinaceous or saccharidic) are, we observe by experience that this often results in a better discrimination of discrete bands than any gel staining. Using this property, we have set up a two-step procedure for obtaining large amount of shell proteins: a blind fractionation of molluscan shell matrices on a preparative denaturing gel electrophoresis, and the detection of the eluted proteins by dot-blot. This approach permits us to acquire structural information on the isolated proteins and to determine their putative functions in biomineralization.

## MATERIALS AND EQUIPMENT

Acetic acid 100% (Merck)
Laemmli sample buffer (Bio-Rad, ref. 161-0737)
Tris buffered saline (10 mM Tris, 0.9% NaCl, pH 7.5)
Electrophoresis buffer (25 mM Tris, 192 mM Glycine, 0.1% w/v SDS, pH 8.3)
CDP-Star, ready-to-use (Roche, ref. 2 041 677)
Gelatin (Calbiochem, ref. 345808)
Tween 20 (Sigma, ref. P-1379)

Fritsch Pulverisette crusher
Titrimeter
Centrifuge
Nalgene filtration assembly
Stirred cell Amicon 400 ml (Millipore, ref. 5124) plusYM10 filters (Millipore, ref. 13642)
Freeze-drying apparatus
Bio-Rad model 491 Prep Cell (ref. 170-2927), including the variable speed cooling pump, the Econo pump, and the model 2110 fraction collector (the Econo UV monitor and the model 1327 chart recorder are not strictly needed)
Bio-Rad Bio dot apparatus (ref. 170-3938)
Immobilon-P transfer membrane (Millipore, ref. IPVH00010)
Dialysis cassette (Pierce) or dialysis tubing
Mini-SDS-PAGE (Protean 3, Bio-Rad, ref. 165-3301) + Mini Trans Blot module (Bio-Rad, ref. 170-3935)

## METHOD

### Shell Matrix Extraction and Polyclonal Antibody Production

The extraction is performed at 4˚C.

1. Crush cleaned shell fragments (bleach-treated) under liquid nitrogen.
2. Suspend the powder (5 to 100 g) in milli-Q water, in a beaker.
3. Add progressively cold acetic acid (5 to 20% vol/vol), until pH 4. The decalcification is controlled by a titrimeter. The decalcification is over when the pH does not vary (overnight decalcification).
4. Centrifuge the solution 10 to 15 min at 5000*g*.
5. Filter the supernatant on a 0.45-μm filter, and discard the pellet.

6. Reduce the volume of the solution by ultrafiltration (Amicon stirred cell, cutoff 10 kDa) to 10 to 30 ml.
7. Dialyze the solution against milli-Q water (several water changes).
8. Freeze dry the solution. Expect 0.02 to 0.3% of the initial weight of the powder.
9. Prepare a polyclonal antibody, by injecting an emulsion containing the antigen and the Freunds adjuvant, in a rabbit (see Note 1).

### Matrix Fractionation of a Preparative Denaturing SDS-PAGE

1. Resuspend and heat denature the freeze-dried acetic acid–soluble matrix in standard Laemmli buffer[9].
2. Cast a preparative gel (see Note 2), according to the manufacturer's specifications[10].
3. Run the sample at constant power (12 W).
4. When the migration front is eluting (after 3.5 h, approx.), start to collect fractions (5 ml per fraction, flow rate 0.5 ml/min).

### Dot-Blot of the Fractions

1. Dot-blot the 80 fractions with the Bio-Dot apparatus, on a Immobilon-P membrane.
2. Block the membrane with 1% gelatin/TBS.
3. Incubate the membrane with the polyclonal antibody diluted in 1% gelatine/TBS/Tween, for 90 min.
4. Rinse the membrane 3 × 10 min with TBS/Tween.
5. Incubate the membrane with the second antibody (GAR, AP conjugate, ref. Sigma A6154) diluted 30,000× in 1% gelatine/TBS/Tween, for 60 min.
6. Rinse the membrane 3 × 10 min. with TBS/Tween.
7. Incubate the membrane in CDP-Star (chemoluminescent substrate) for few minutes.
8. Expose the membrane to a film (Kodak X-Omat), and develop it.

### Test of the Fractions on a Mini-SDS-PAGE

1. Following the results, pool the tubes of interest (see Note 3). To reduce the volume of the pooled tubes, use the Amicon ultrafiltration cell.
2. Extensively dialyze the fractions against milli-Q water for several days in a dialysis cassette or tube. Change the water several times.
3. Freeze dry the fractions.
4. Check the purity of the fractions on a mini-SDS-PAGE, with silver nitrate staining[11].

## NOTES

1. A standard immunization procedure is performed with injections at 0, 14, 28, and 56 days, and bleedings at 0 (preimmune), 38, 66, and 80 days. The respective titers of the collected antisera are determined by ELISA, and their specificity is verified on western-blots. Because the antigen (the shell matrix) is a mixture of glycoproteins and polysaccharides, the resulting antibodies may be raised against proteinaceous and saccharidic epitopes. This does not have any influence on the subsequent preparative fractionation. A way to determine the nature of the epitope consists in degrading the matrix with proteinase K (proteinaceous epitopes) or with Na-periodate (saccharidic epitopes), and to measure, by ELISA or dot-blot, the loss of reactivity of the treated matrix.

2. A 10 to 12% acrylamide gel is prepared according to a standard procedure[10]. During polymerization, the separation gel is cooled down by circulating water in the cooling core. Polymerization is performed overnight. The volume used for the stacking gel should be at least twice that of the sample. The elution buffer (Tris/glycine) has the same molarity as the running buffer, but does not contain SDS.
3. Because the shell matrix is a mixture of components, which exhibit very different immunogenicities, the antiserum used does not permit us to quantify specific bands on Western-blots nor on dot-blots. Quantification may be performed from the freeze-dried fractions, by weighing the lyophilisates or by redissolving them and performing a micro-BCA.

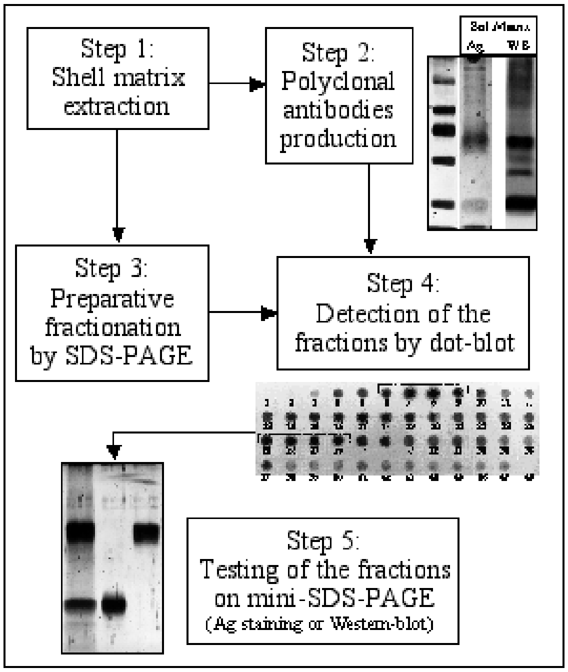

**FIGURE 1.** A general strategy for successfully purifying large amount of molluscan shell proteins, illustrated with the example of the shell matrix of the bivalve *Pinna nobilis*. After the extraction (step 1), the shell matrix is used for generating polyclonal antibodies (step 2), which are tested on western-blots (lane WB) against the soluble matrix. The shell proteins are subsequently fractionated on a preparative electrophoresis (step 3), and detected by dot-blot with the polyclonal antibody (step 4). The purity of the fractions can be checked on mini-SDS-PAGE (step 5). Note that the staining of the matrix on western-blot (lane WB) gives more sharp bands than the silver staining (lane Ag, step 2).

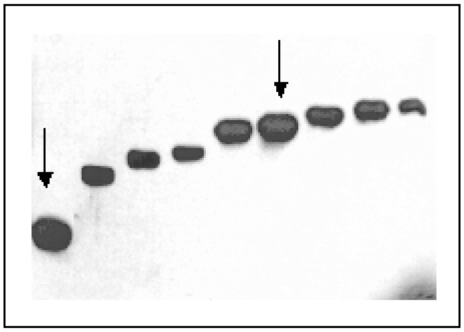

**FIGURE 2.** Western-blot of fractions of the shell matrix of *Pinna nobilis*. The arrows indicate the two main fractions, which are also visualized in Fig. 1. Some minor and less immunogenic fractions have also been collected. The fact that these fractions are visualized on the final western-blot and not on the initial blot of the whole matrix (lane WB, Fig. 1, step 2) is explained by a "concentration" effect. On the final blot, the fractions are much more concentrated, and are subsequently detected by the antimatrix serum, in spite of their low immunogenicity

## ACKNOWLEDGMENTS

This work was supported by Fondation Simone et Cino Del Duca (Paris, France) for the period November 1999–January 2001, and by IsoTis, for the period February 2001–December 2002.

## BIOSKETCH

**Frédéric Marin, Ph.D.** is a research scientist with 10 years postdoctoral experience in the fields of biochemistry of calcified tissues, immunology, and molecular biology. His research interests include the molecular aspects of molluscan shell formation, genetics of invertebrate calcified systems, and the origin of metazoan mineralization.
